# TrpC3 Regulates Hypertrophy-Associated Gene Expression without Affecting Myocyte Beating or Cell Size

**DOI:** 10.1371/journal.pone.0000802

**Published:** 2007-08-29

**Authors:** Jacob S. Brenner, Ricardo E. Dolmetsch

**Affiliations:** 1 Program in Chemical and Systems Biology, Stanford University, Stanford, California, United States of America; 2 Department of Neurobiology, Stanford University, Stanford, California, United States of America; Monash University, Australia

## Abstract

Pathological cardiac hypertrophy is associated with an increased risk of heart failure and cardiovascular mortality. Calcium (Ca^2+^) -regulated gene expression is essential for the induction of hypertrophy, but it is not known how myocytes distinguish between the Ca^2+^ signals that regulate contraction and those that lead to cardiac hypertrophy. We used *in vitro* neonatal rat ventricular myocytes to perform an RNA interference (RNAi) screen for ion channels that mediate Ca^2+^-dependent gene expression in response to hypertrophic stimuli. We identified several ion channels that are linked to hypertrophic gene expression, including transient receptor potential C3 (TrpC3). RNAi-mediated knockdown of TrpC3 decreases expression of hypertrophy-associated genes such as the A- and B-type natriuretic peptides (ANP and BNP) in response to numerous hypertrophic stimuli, while TrpC3 overexpression increases BNP expression. Furthermore, stimuli that induce hypertrophy dramatically increase TrpC3 mRNA levels. Importantly, whereas TrpC3-knockdown strongly reduces gene expression associated with hypertrophy, it has a negligible effect on cell size and on myocyte beating. These results suggest that Ca^2+^ influx through TrpC3 channels increases transcription of genes associated with hypertrophy but does not regulate the signaling pathways that control cell size or contraction. Thus TrpC3 may represent an important therapeutic target for the treatment of cardiac hypertrophy and heart failure.

## Introduction

Cardiac hypertrophy is associated with most chronic diseases of the heart, including hypertension, valvular disease, ischemia, and heart failure. While initially believed to be a beneficial adaptation to stress, mounting clinical and experimental evidence indicate that hypertrophy is maladaptive because it predisposes patients to myocardial infarction, lethal arrhythmia, and heart failure (reviewed in [1]). Therefore, cardiac hypertrophy is a major therapeutic target for the treatment of heart disease [2].

Ca^2+^ is a critical second messenger in the signaling pathways leading to hypertrophy [3], and several Ca^2+^-regulated phosphatases, kinases, and transcription factors play essential roles in hypertrophy [4]–[7]. Because Ca^2+^ regulates *both* myocyte contraction and cardiac hypertrophy, a key question is how myocytes distinguish between the Ca^2+^ that causes contraction and the Ca^2+^ that regulates transcription and hypertrophy [8]? One hypothesis is that specificity is achieved by having the contractile and hypertrophy-inducing machinery respond to different features of the intracellular Ca^2+^ signal. According to this kinetic discrimination model, the signaling cascades that lead to hypertrophy are activated when hormonal stimulation changes the kinetics or the amplitude of the Ca^2+^ transients that generate cardiac contraction [9]. Some support for this model comes from observations that perturbations that increase the amplitude, pulse duration, or frequency of the Ca^2+^ elevations that occur during beating all induce mild hypertrophy [10]–[14]. An alternative hypothesis is that Ca^2+^ signals that regulate hypertrophy are spatially segregated from the Ca^2+^ signals that regulate contraction. One way to achieve the spatial separation of Ca^2+^ signals involved in hypertrophy and contraction is by having separate Ca^2+^ channels linked to signaling proteins that regulate either contraction or hypertrophy. The sarcolemmal Ca^2+^ channel involved in contraction, Ca_V_1.2 (an L-type Ca^2+^ channel), is localized to the T-tubules, is closely apposed to the ryanodine receptor RyR2 and other components involved in contraction, and therefore generates localized Ca^2+^ elevations that efficiently activate the contractile apparatus. Thus far, however, the identity and localization of the channels that regulate gene expression and induce hypertrophy are not known.

Indirect evidence in favor of the spatial segregation model comes from studies of G-protein coupled receptors (GPCRs) in the heart. Cardiac hypertrophy is induced by activation of several GPCRs, such as the α-adrenergic receptor, the angiotensin II receptor, and the endothelin receptor (reviewed in [15]). GPCRs stimulate a variety of ion channels by activating phospholipase C (PLC) and other enzymes. Pharmacological data suggests that myocytes possess GPCR-stimulated calcium currents that are independent of Ca_V_1.2 [16], [17]. Furthermore, pharmacological blockade of such currents has a greater effect on a variety of hypertrophy phenotypes than does blockade of Ca_V_1.2 [16]. These results suggest that GPCR-stimulated hypertrophy employs ion channels distinct from Ca_V_1.2.

We report here our initial attempts to identify the ion channels that regulate the induction of the gene expression program associated with cardiac hypertrophy. We employed two unbiased methods to identify ion channels that regulate cardiac hypertrophy: a real-time PCR-based assay for ion channels that are expressed and dynamically regulated during hypertrophy, and an RNA interference screen to search for channels that are necessary for cardiac hypertrophy. Our search initially focused on the Group 1 transient receptor potential (Trp) superfamily of ion channels. The Group 1 Trp channels, are a family of six-transmembrane-domain cation channels with varying degrees of Ca^2+^ selectivity (reviewed in [18]) that include the TrpC, TrpV, TrpM, and TrpA subfamilies. They share significant sequence similarity and wide tissue distribution, and are only distantly related to the Group 2 Trp channels (TrpML and TrpP subfamilies). The Group 1 Trp channels are good candidates for a role in hypertrophy, as many are expressed in the heart [19], [20] and previous work in other cell types has shown that Trp channels are activated by many of the same GPCRs that induce hypertrophy in cardiac myocytes [21]–[23].

Our screens identified TrpC3 as a mediator of cardiac hypertrophy. TrpC3 mRNA levels are highly upregulated by hypertrophic stimuli. In α_1_-adrenergic stimulated myocytes, TrpC3 knockdown decreases hypertrophic gene expression while TrpC3 overexpression augments the expression of hypertrophy marker genes. However, TrpC3 knockdown does not affect cell size. Intriguingly, the intracellular localization of TrpC3 channels is quite distinct from that of Ca_V_1.2 and RyR2. TrpC3's localization and lack of effect on beat frequency and beating-associated calcium suggests that it may produce calcium signals that are spatially independent of those involved in contraction. These results identify an ion channel that plays a prominent role in the induction of hypertrophy and which may be a promising target for the treatment of heart disease.

## Results

### RNAi screen identifies TrpC3 as an ion channel involved in cardiac hypertrophy

**Figure 1:**
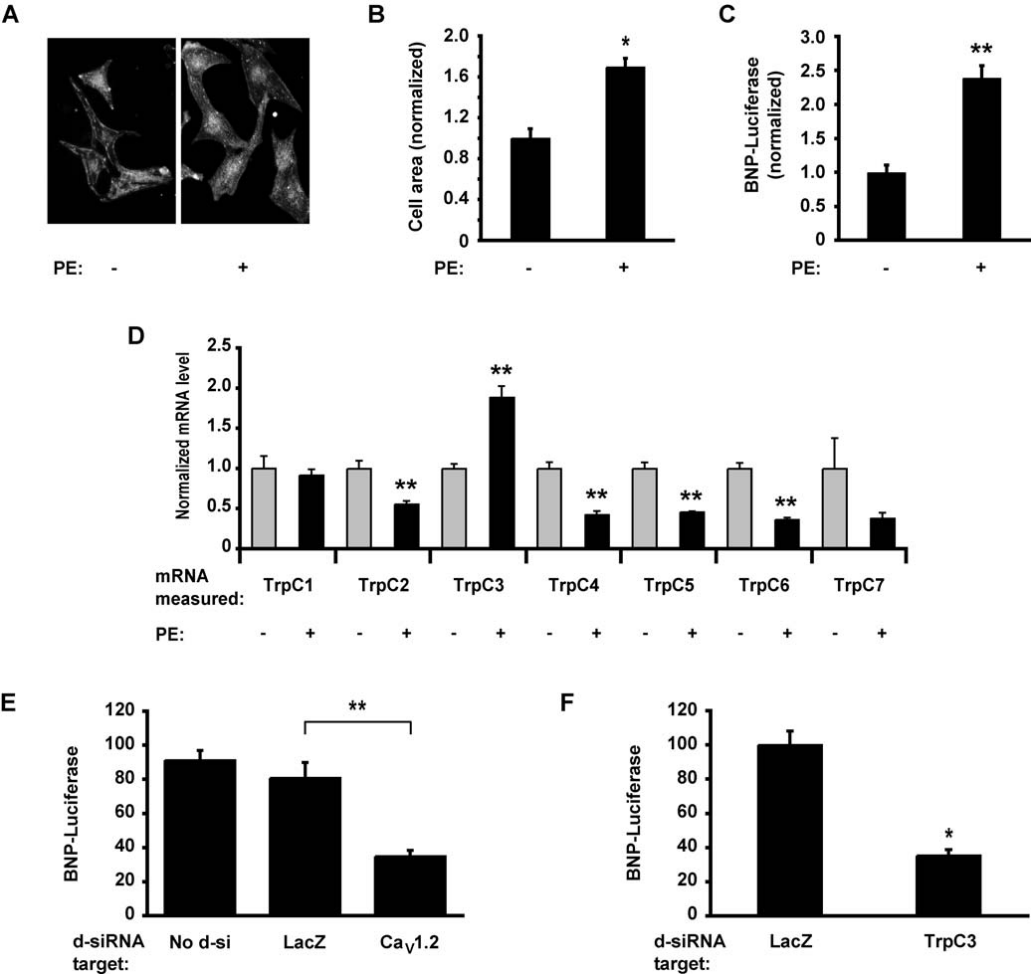
RNAi screen identifies TrpC3 as a mediator of hypertrophy-associated gene expression. (*A*) Increase in cell size in neonatal rat ventricular myocytes stained with anti-α-actinin either untreated (*left panel*) or stimulated with 20 µM PE for 60 hours (*right panel*) to induce hypertrophy. (*B*) Quantification of the two-dimensional cell area for the experiment described in *A*. Data are normalized to the mean of the cell-area for untreated cells. For each condition, an average of n = 28 cells were analyzed. (*C*) Luciferase expression in myocytes transfected with the BNP-luciferase (BNP-Luc) reporter gene in the absence (*left bar*) or presence (*right bar*) of 20 µM PE for 60 hours. BNP-luciferase activity is normalized to the activity of a co-transfected plasmid constitutively expressing *Renilla* luciferase. n = 4 biological replicates per condition. (*D*) Real-time RT-PCR measurements of TrpC mRNA expression in untreated myocytes (*grey bars*) or myocytes stimulated with 20 µM PE for 60 hours (*black bars*). The data is normalized to the mean of the untreated myocytes for each TrpC gene. Asterisks indicate a significant difference between untreated and PE-stimulated cells. n = 4 measurements per condition. (*E*) Luciferase activity measured in myocytes transfected with BNP-Luc and d-siRNA targeting either the LacZ or Ca_V_1.2 genes and stimulated with PE. Ca_V_1.2 d-siRNA reduces the expression of the BNP-Luc reporter gene. n = 6 biological replicates per condition. (*D*) Ventricular myocytes were transfected with d-siRNAs targeting twenty of the twenty-two rat TRP channels along with BNP-Luc, stimulated with 20 µM PE for 48 hours and assayed luciferase activity. The luciferase activity of cells containing d-siRNAs targeting LacZ and the best hit, TrpC3 are shown. n = 12 biological replicates per condition. * indicates statistically significant difference compared to control, P<0.0001. ** indicates P<0.01. For all bar graphs, data are represented as mean±SEM.

To identify Ca^2+^ channels that are important for the induction of cardiac hypertrophy, we first measured the expression of candidate ion channels in resting and hypertrophic cells. We stimulated neonatal rat ventricular myocytes with the α_1_-adrenergic agonist phenylephrine (PE) for 60 hours to induce hypertrophy. Stimulation with PE led to a 69%±16% increase in cell size (Fig. 1*A* and1*B*) and to a 138±20% increase in the activity of the promoter of BNP (Fig. 1*C*), a gene critically involved in the pathology of cardiac hypertrophy (we use the term “BNP” to refer to the gene alternately called “pre-pro-BNP” or “Nppb”). We next used real-time RT-PCR to measure expression at the mRNA level of Group 1 Trp channels in both resting and hypertrophic cardiac myocytes. We found that all the TrpC channels were significantly expressed in ventricular myocytes and that several were dynamically regulated during hypertrophy. One channel, TrpC3, was significantly upregulated in hypertrophic myocytes (an increase of 89%±15%), whereas most of the other TrpC channels were down-regulated by treatment with PE (Fig. 1*D*). These results show that many Group1 Trp channels are expressed in myocytes and are regulated by the signaling pathways that lead to hypertrophy.

Because many Group1 Trp channels are dynamically regulated during hypertrophy, we next investigated which of these channels are functionally important for this process. We designed an RNAi screen using *in vitro*-diced double-stranded RNAs (d-siRNA) to reduce the expression of the Group 1 Trp channels in rat ventricular myocytes *in vitro*. We used d-siRNAs because this approach has been reported to increase the efficiency of gene suppression and to reduce off-target effects in other systems [24]. We introduced the d-siRNAs into cardiac myocytes along with a reporter plasmid containing luciferase under the control of the BNP promoter (BNP-Luc). We then stimulated the cells with PE for 48 hours, after which we measured luciferase activity in cell lysates using a 96-well automated luminometer. Before conducting our full screen we performed a pilot experiment by transfecting myocytes with BNP-Luc and d-siRNA targeting the Ca_V_1.2 gene or the bacterial gene LacZ. As expected, the d-siRNA against LacZ had no effect on BNP-Luc, but the d-siRNA against Ca_V_1.2 dramatically reduced BNP-Luc activity (Fig 1*E*). This is consistent with previous results suggesting that Ca^2+^ influx and contraction mediated by Ca_V_1.2 are necessary for the induction of cardiac hypertrophy. In addition, this result provides proof-of-principle that this assay is a viable method for identifying ion channels that regulate cardiac hypertrophy.

We next extended our screen to cover all the Group 1 Trp family members. We generated twenty d-siRNAs targeting all the Group1 Trp channels, except TrpM1 and TrpM5 which could not be amplified from myocyte mRNA. d-siRNAs targeting three Trp family members, including TrpC3, strongly reduced the expression of BNP-Luc. TrpC3 d-siRNA decreased the activity of BNP-Luc by 64.6%±9.8% (Fig. 1*F*). This decrease in BNP-Luc expression was repeated in multiple experiments using different concentrations of TrpC3 d-siRNA and different times of incubation, suggesting that TrpC3 plays an important role in activating the signaling cascades necessary for BNP production in hypertrophic cardiac myocytes.

### TrpC3 is necessary for hypertrophic gene expression

**Figure 2:**
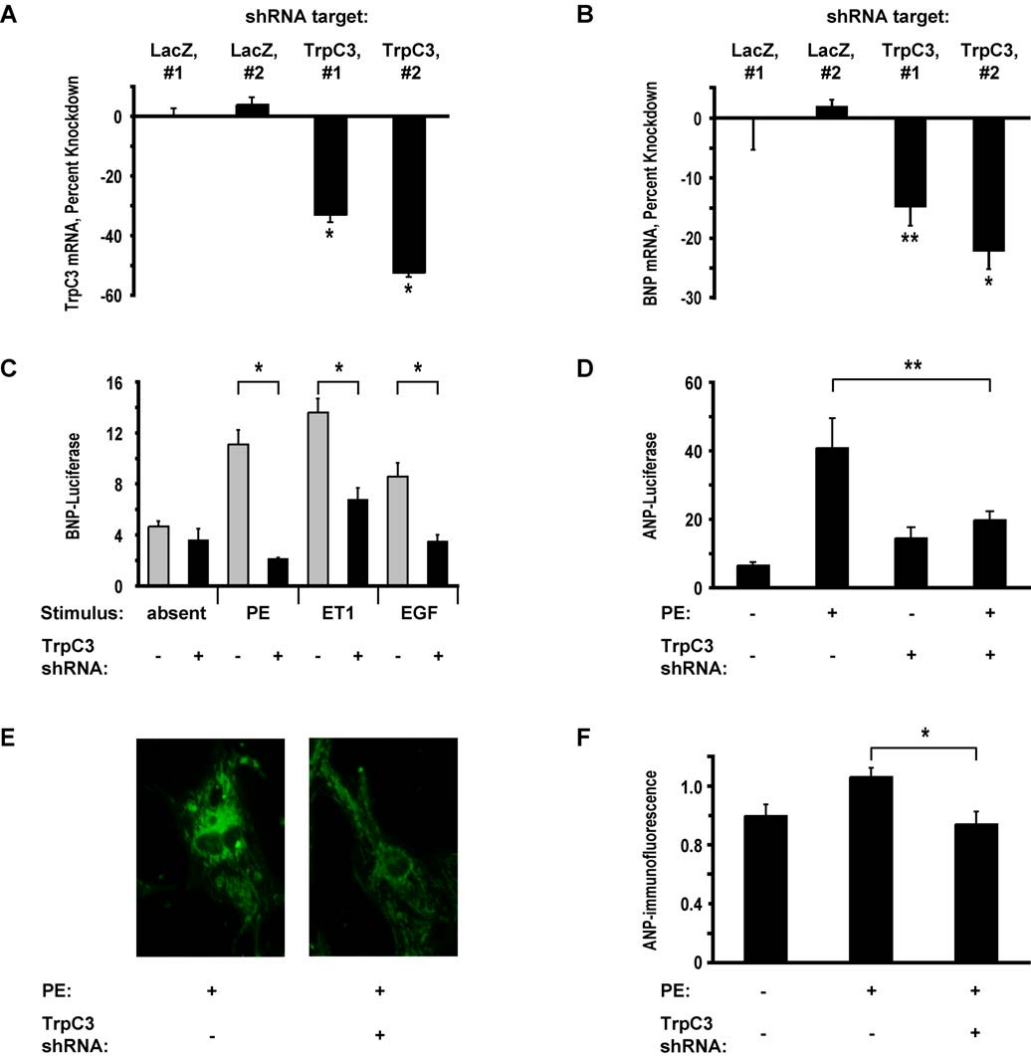
TrpC3 is necessary for hypertrophic gene expression. (*A*) Real-time RT-PCR measurement of TrpC3 mRNA in myocytes infected with lentiviruses expressing shRNAs targeting either LacZ or TrpC3. Data is presented as percent decrease in expression compared to the mean expression of myocytes expressing LacZ-shRNA#1. Asterisks indicate significant difference compared to LacZ-shRNA#1. n = 4 per condition. (*B*) Real time RT-PCR measurements of BNP mRNA levels in myocytes infected with the same lentiviruses as in *A*. TrpC3 shRNAs reduce the expression of endogenous BNP mRNA. n = 4 per condition. (*C*) Luciferase activity in myocytes transfected with BNP-Luc and plasmids expressing shRNAs targeting either TrpC3 or LacZ. Cells were stimulated for 60 hours with 20 µM phenylephrine (PE), 100 nM endothelin-1 (ET1) or 150 ng/mL epidermal growth factor (EGF) to induce hypertrophy. n = 4 biological replicates per condition. (*D*) Luciferase expression in myocytes transfected with ANP-Luc and untreated or stimulated with 20 µM PE. n = 6 biological replicates per condition. (*E*) Myocytes transfected with plasmids expressing shRNAs targeting either LacZ or TrpC3 were stimulated with 20 µM PE for 60 hours and then fixed and immunostained with an anti-ANP antibody. TrpC3 shRNA expression caused a decrease in ANP staining. (F) Quantification of immunostained cells treated as in *E*. The integrated ANP-immunofluorescence intensity across the two-dimensional projection of transfected cells was measured. Average n = 31 cells per condition. In all panels, cells not expressing TrpC3-shRNA were expressing a control shRNA against LacZ. * indicates statistically significant difference compared to control, P<0.01. ** indicates P<0.05. For all bar graphs, data are represented as mean±SEM.

To verify that the effects of the TrpC3 d-siRNA pools are not a consequence of off-target effects, we next used a different technique for reducing the expression of TrpC3 in myocytes. We generated lentiviruses encoding short-hairpin RNAs (shRNAs) targeting TrpC3, as this method has a different set of off-target effects than the d-siRNA pools used in the initial screen above. We designed two shRNAs to target different sequences of TrpC3 (TrpC3-shRNA-1 and -2) and two shRNAs targeting the bacterial gene LacZ as a negative control (LacZ-shRNA-1 and -2). We packaged the shRNA vectors into lentiviral capsids, infected ventricular myocytes, and cultured the cells for four days. We first verified that the lentiviral shRNAs reduced the expression of TrpC3 using real-time RT-PCR to measure endogenous TrpC3 transcripts. Compared to myocytes expressing shRNAs targeting LacZ, myocytes expressing TrpC3-shRNA had significantly lower TrpC3 mRNA levels (Fig 2*A*). As only ∼65% of myocytes were infected by the lentiviruses, the 52.4%±4.0% decrease in expression measured at the population level implies a 70% knockdown of TrpC3 mRNA in individual infected cells. We next measured the expression of endogenous BNP mRNA in the infected cells. As shown in Figure 2*B*, myocytes expressing TrpC3-shRNA have significantly lower levels of BNP mRNA relative to myocytes expressing the control LacZ-shRNA. These experiments demonstrate that two different TrpC3-shRNAs decrease BNP expression and provide strong evidence that TrpC3 is necessary for the expression of BNP in hypertrophic myocytes.

Cardiac hypertrophy can be induced by treating ventricular myocytes with a wide variety of stimuli, ranging from GPCR agonists like endothelin-1 (ET1) and PE to receptor tyrosine kinase agonists such as epidermal growth factor (EGF). To determine which types of stimuli require TrpC3 channels to induce hypertrophy, we introduced the TrpC3 shRNAs and BNP-Luc into cardiac myocytes and stimulated the cells with either PE, ET1, or EGF. All of these agonists induced a significant induction of BNP expression that was greatly diminished in cells expressing a TrpC3-shRNA (Fig. 2*C*). Intriguingly, we found that TrpC3-shRNA does not cause a significant decrease in BNP-luciferase activity in cells that are not exposed to a hypertrophic stimulus (Fig. 2*C*). These results suggest that TrpC3 is a common element in multiple pathways leading to the induction of cardiac hypertrophy and that it plays only a minor role in regulating BNP levels in non-hypertrophic ventricular myocytes.

We next investigated whether TrpC3 is essential for the induction of not only BNP, but also of other genes associated with hypertrophy. ANP plays an important role in the pathology of cardiac hypertrophy and heart failure and is significantly upregulated in hypertrophic myocytes. To determine if TrpC3 is important for the expression of the ANP gene, we first measured the activity of an ANP-Luciferase reporter gene (ANP-Luc) in cells expressing TrpC3-shRNAs. While treatment with PE increased the expression of ANP-Luc more than 6-fold in cells expressing a control shRNA, PE caused only a 1.4-fold increase in ANP-Luc expression in cells expressing TrpC3-shRNA (Fig. 2*D*). To determine if TrpC3 is necessary for the expression of endogenous ANP, we measured ANP protein levels using quantitative immunocytochemistry with an anti-ANP antibody. As expected, ANP protein levels increased upon PE stimulation, but importantly, they were dramatically reduced in cells expressing TrpC3-shRNA (Fig. 2*E and 2F*). Taken together these results indicate that TrpC3 plays an important role in the induction of both ANP and BNP, the two best-studied cardiac hypertrophy genes.

### Increased TrpC3 levels augment hypertrophic gene expression

**Figure 3:**
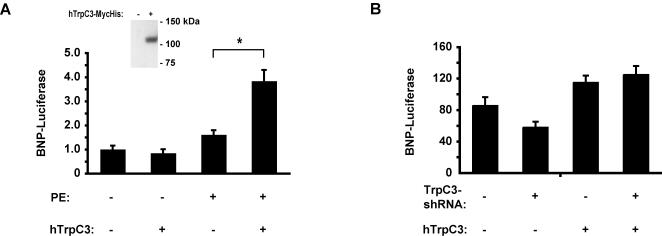
TrpC3 is sufficient for hypertrophy-associated gene expression. (*A*) Luciferase expression in myocytes co-transfected with BNP-Luc and either a plasmid expressing hTrpC3 or the empty parent plasmid. Cells were stimulated for 60 hours with 20 µM phenylephrine (PE), or left un-stimulated for the same period. *Inset*: Western blot analysis of HEK-293T cells transfected either with the parent plasmid or a plasmid expressing hTrpC3 C-terminally tagged with Myc and 6x-His epitopes. The band shown is ∼100 kDa. (*B*) Luciferase measurements of myocytes containing plasmids encoding shRNAs targeting TrpC3 or LacZ and expression plasmids expressing hTrpC3 or an empty vector. hTrpC3 expression prevents the reduction of BNP expression caused by TrpC3-shRNA expression. For both *A* and *B*, n = 6 biological replicates per condition. * indicates statistically significant difference compared to control, P<0.005. For all bar graphs, data are represented as mean±SEM.

The experiments above suggest that TrpC3 expression is *necessary* for the expression of both ANP and BNP, two genes that play an important role in cardiac hypertrophy. To determine if the expression of TrpC3 is *sufficient* for the production of ANP or BNP, we overexpressed TrpC3 in cardiac myocytes and measured the activation of the BNP-Luc reporter gene. Introduction of a plasmid encoding human TrpC3 (hTrpC3) into cells led to the expression of a protein of approximately 100 kDa, as expected (Fig. 3*A*, *inset*). Expression of hTrpC3 in myocytes that were not stimulated with PE had no effect on BNP promoter activity (Fig. 3*A* *,* comparing *columns 1* & *2*). Thus increasing TrpC3 levels alone is not sufficient to induce hypertrophic gene expression. However, in cells stimulated with PE for 60 hours, overexpression of hTrpC3 caused a greater than two-fold increase in the expression of the BNP-Luc reporter gene (Fig. 3*B* *,* comparing *columns 3* & *4*). These results suggest that overexpression of TrpC3 strongly augments BNP gene expression, but that this effect depends upon co-stimulation with hypertrophic agonists such as PE.

We next employed hTrpC3 overexpression to determine if the effects of the TrpC3-shRNAs on the expression of BNP are due to the specific suppression of TrpC3 or are due to off-target effects. We expressed hTrpC3, which is insensitive to rat TrpC3-shRNA, in the presence of rat TrpC3-shRNA and subsequently measured BNP-Luc activity. In the absence of hTrpC3, rat TrpC3-shRNA caused a decrease in BNP-Luc activity (Fig. 3*B*, comparing *columns 1* & *2*). The reduction in BNP-Luc was smaller than we had observed previously (Fig 2C), because we co-expressed the TrpC3-shRNA with plasmids containing the CMV promoter. The CMV promoter reduces the expression from other plasmid promoters leading to reduced expression of TrpC3-shRNA. Importantly, hTrpC3 expression rescued the effect of the rat TrpC3-shRNA on BNP-Luc activity (Fig. 3*B*, comparing *columns 3* & *4*). This result indicates that TrpC3-shRNA suppressed BNP-Luc by specifically suppressing the expression of the endogenous rat TrpC3 and not by suppressing other genes.

### TrpC3 does not regulate cell size

One of the hallmarks of cardiac hypertrophy is an increase in myocyte size. We therefore examined whether TrpC3 is necessary for the increase in cell size associated with cardiac hypertrophy. To do this, we developed a high-throughput assay of myocyte size that would allow for rapid screening of the effects of knocking down TrpC3 and other candidate hypertrophy genes identified in our screens. Myocytes were transfected with plasmids that co-express DsRed along with an shRNA that reduces the expression of the target gene. After 48 hours of PE stimulation, the cells were fixed and stained with anti-α-actinin antibody to distinguish myocytes from other cell types in the cultures. Because myocytes have low transfection efficiencies and variable size, we used an automated microscope to collect images of large numbers of cells (more than 1000 transfected myocytes per condition) to achieve greater statistical power than traditional manual measurements of cell size. We analyzed these images using an automated analysis program that identified cells expressing DsRed, determined if they were myocytes based on the intensity of anti-α-actinin staining, and then measured cell size.

**Figure 4:**
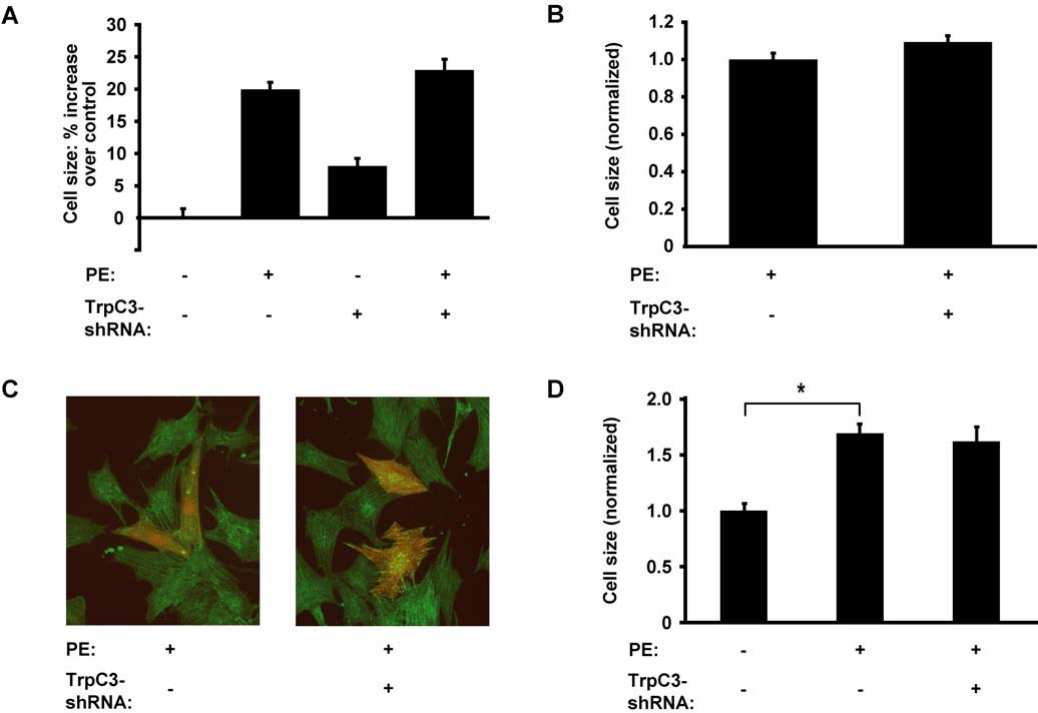
Reduction of TrpC3 does not prevent the increase in cell size associated with hypertrophy. (*A*) Cell size measurement of ventricular myocytes expressing either TrpC3 or a negative control shRNA and stimulated with 20 µM PE for 48 hours. Size is shown as percent increase compared to the size of unstimulated myocytes expressing negative control shRNA (the *left column*). Cell size was measured using an automated fluorescence microscope and analysis program. For each condition, n >1000 transfected myocytes analyzed. *Columns 2–4* were each significantly different than *column 1*, P<0.001. *Columns 2* and *4* (PE-stimulated myocytes expressing negative control shRNA or TrpC3-shRNA, respectively) are not significantly different from each other. (*B*) The results in *A* were confirmed by using manual-measurements of myocyte cell size in cells stimulated with 20 µM PE for 60 hours. Cells expressing TrpC3-shRNA were not significantly different in size than those expressing LacZ-shRNA. n = 125 cells per condition. (*C*) Examples of myocytes expressing negative control shRNA (left) and TrpC3-shRNA (right) used in the manual measurement of cell size. Myocytes were stained with anti-α-actinin antibody (green), and cells that were expressing shRNAs are red. (*D*) Myocytes expressing LacZ- or TrpC3-shRNAs were stimulated with 20 µM PE for 60 hours and then fixed, immunostained with anti-ANP antibody, and measured simultaneously for cell size and ANP-immunofluorescence. TrpC3-shRNA had no effect on cell size (*columns 2* and *3*), but significantly decreased ANP expression (data displayed in Fig. 2*F*). Average n = 31 cells per condition. * indicates statistically significant difference compared to control, P<0.0001. For all bar graphs, data are represented as mean±SEM.

To determine the effects of TrpC3 on cell size, we used the high-throughout cell size assay to measure the effects of PE stimulation and TrpC3-shRNA. Stimulation with PE caused a 20%±1.1% increase in rat ventricular myocyte cell size, as measured in more than 1000 cells per condition. The magnitude of this effect is similar to what has been observed using other techniques to measure increases in cell size. We next introduced TrpC3-shRNA into these cells to reduce the expression of TrpC3. Surprisingly, TrpC3-shRNA had no effect on the increase in cell size caused by PE stimulation (Fig. 4*A columns 2* vs *4*), but did cause a small increase in cell size in unstimulated cells. The lack of effect of TrpC3-shRNA in PE-stimulated cells was confirmed by using the manual method of cell size measurement on a smaller number of myocytes (125 cells per condition) stimulated with or without PE for 60 hours (Fig. 4*B*; representative myocytes transfected with negative control shRNA and TrpC3-shRNA are shown in Fig. 4*C* *left* and *right panels*, respectively). We repeated these experiments using myocytes infected with lentiviruses driving expression of TrpC3-shRNA and found no effect on the increase in myocyte size after PE stimulation. Additionally, we measured both cell size and endogenous ANP expression simultaneously in the same PE-stimulated cells, and found that TrpC3-shRNA decreased ANP expression (Fig. 2*F*), but had no effect on cell size (Fig. 4*D*). Thus we conclude that while TrpC3 expression is necessary for the expression of ANP and BNP, it has little or no effect on the increase in cell size associated with cardiac hypertrophy.

### TrpC3 regulates resting Ca^2+^ and PE-induced Ca^2+ ^elevation but not beating

**Figure 5:**
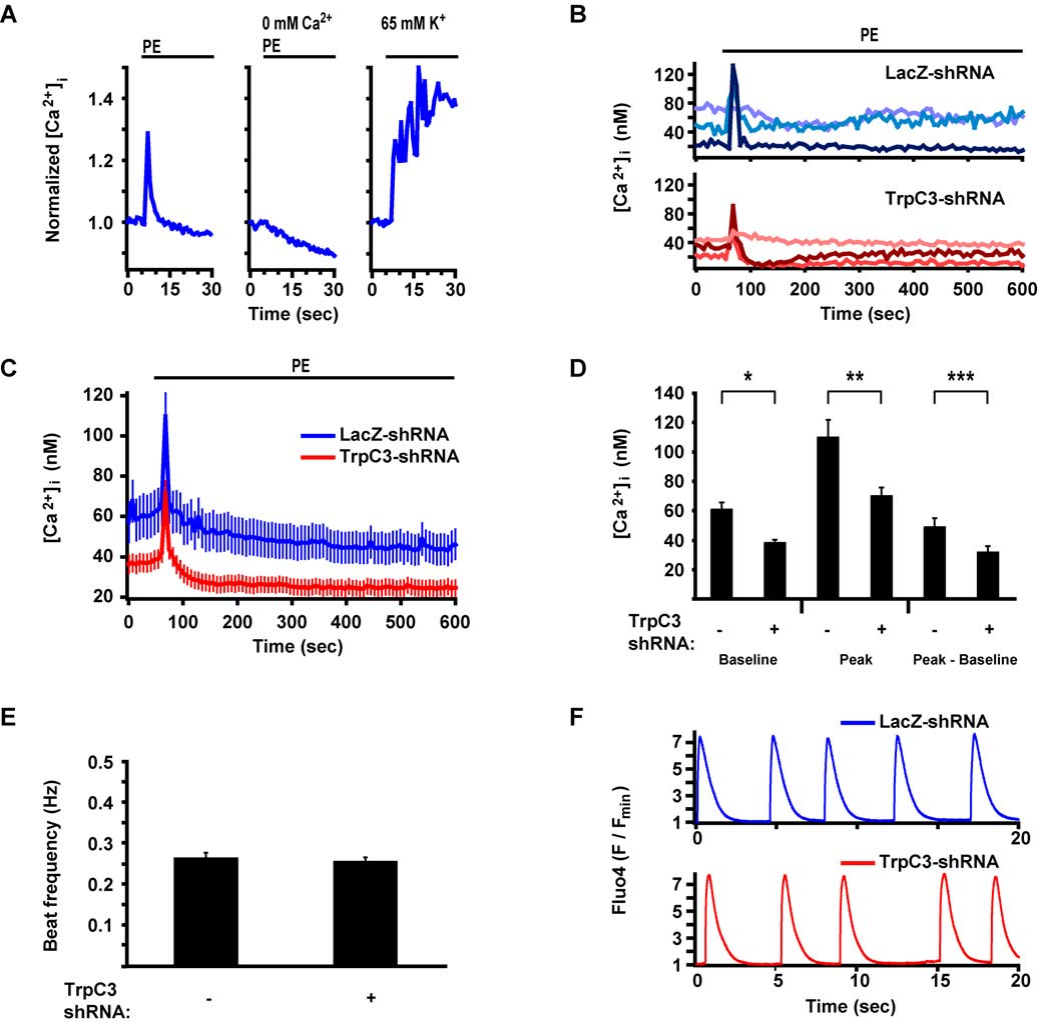
TrpC3 regulates baseline [Ca^2+^]_i_ and the PE-induced [Ca^2+^]_i_ elevation but does not affect beat frequency. (*A*) Mean [Ca^2+^]_i_ (normalized) obtained by fura-2 measurements in uninfected myocytes. Myocytes maintained in media containing 2 mM Ca^2+^ and the Ca_V_1.2 blocker verapamil were then stimulated with 20 µM PE (in addition to verapamil) in the presence of 2 mM extracellular Ca^2+^ (*left panel*, n = 58 cells) or 0 mM extracellular Ca^2+^ (*center panel*, n = 101 cells) for the time indicated by the bar at the top of the panels.. *Right panel* (n = 58 cells), myocytes previously stimulated with PE and incubated in media containing 2 mM Ca^2+^ and verapamil were then perfused with depolarizing media containing 65 mM K^+^ and no verapamil. (*B-C*) [Ca^2+^]_i_ measurements in myocytes infected with lentiviruses expressing shRNAs targeting LacZ or TrpC3. Ratiometric fura-2 [Ca^2+^]_i_ measurements were performed on myocytes treated with verapamil and then stimulated with 20 µM PE (in addition to verapamil) for the time indicated by the bar at the top of the panels. Representative traces from single myocytes are shown in *B (top panel* depicts examples of individual cells expressing LacZ-shRNA; *bottom panel*, TrpC3-shRNA). Average traces are shown in *C* and represent the mean of 82 cells expressing LacZ-shRNA (*blue*) and 117 cells expressing TrpC3-shRNA (r*ed*). (*D*) Mean baseline, peak, and peak minus baseline [Ca^2+^]_i_ of cells depicted in *C*. Average baseline [Ca^2+^]_i_ sampled at 5 time-points before treatment with PE (*columns 1* and *2*), average amplitude of the [Ca^2+^]_i_ peak triggered by PE stimulation (*columns 3* and *4*), and peak minus baseline calculated for each individual myocyte and then averaged across the population (*columns 5* and *6*). (*E*) Spontaneous beat frequency of myocytes infected with lentiviruses expressing either LacZ- or TrpC3-shRNAs and stimulated for 48 hours with PE to induce hypertrophy. Beat frequency was measured by phase contrast microscopy in 20 wells per condition. TrpC3 shRNAs had no effect on myocyte beat frequency. (*F*) Fluo-4 measurements of [Ca^2+^]_i_ in spontaneously-beating myocytes expressing LacZ- or TrpC3-shRNAs and stimulated for 72 hours with PE. Calcium traces are displayed as Fluo-4 intensity values normalized by the minimum Fluo-4 value recorded from the given cell. Displayed traces are from single representative myocytes expressing either LacZ-shRNA (*top panel*) or TrpC3-shRNA (*bottom panel*). Comparing Fluo-4 calcium traces for myocytes expressing LacZ-shRNA (n = 23 cells) and TrpC3-shRNA (n = 23 cells) did not detect any statistical difference in beat frequency, peak:baseline ratio, or decay constants. * indicates statistically significant difference compared to control, P<0.0001. ** indicates P<0.005. For all bar graphs and the traces in *B*, data are represented as mean±SEM.

Since gene expression associated with hypertrophy is regulated by Ca^2+^, we next sought to determine the role of TrpC3 in regulating intracellular Ca^2+ ^([Ca^2+^]_i_). We began by examining the effect of PE stimulation on [Ca^2+^]_i_, because we had found that TrpC3 knockdown abrogated PE-induced gene expression. We loaded uninfected myocytes with Fura-2 and imaged them on a digital-imaging microscope to record [Ca^2+^]_i_ levels. To observe the effect of PE on [Ca^2+^]_i_ independently of beating and of Ca^2+^ influx through Ca_V_1.2, we pre-incubated and imaged the cells in media containing the Ca_V_1.2-blocker verapamil. Stimulation with 20 µM PE in media containing 2 mM Ca^2+^ and verapamil caused a transient [Ca^2+^]_i_ elevation that lasted approximately ten seconds and a subsequent [Ca^2+^]_i _decline that lasted at least 600 seconds (Fig. 5*A*, *left panel*). However, when the myocytes were stimulated with PE in calcium-free media, the [Ca^2+^]_i_ elevation was greatly diminished (Fig. *5A*, comparing *left* and *center panels*). This suggests that the PE-induced [Ca^2+^]_i _elevation requires extracellular calcium. We next compared the PE-induced [Ca^2+^]_i_ elevation to the [Ca^2+^]_i_ rise produced by depolarizing myocytes. Myocytes were incubated in media containing 2 mM Ca^2+^ and verapamil and stimulated with PE for 3 minutes, by which time their [Ca^2+^]_i_ had reached a plateau. The myocytes were then perfused with media containing 65 mM K^+^ and no verapamil, which induced a [Ca^2+^]_i_ elevation (Fig. 5*A*, *right panel*) that was greater in amplitude and sustained much longer than the transient [Ca^2+^]_i _elevation triggered by PE. Thus the PE-induced [Ca^2+^]_i_ elevation requires extracellular calcium and is relatively small in magnitude compared to a depolarization-induced calcium rise.

We next examined the effect of TrpC3 knockdown on [Ca^2+^]_i_ immediately before and after stimulation with PE. Myocytes infected with lentiviruses encoding TrpC3-shRNA or a control LacZ-shRNA were loaded with Fura-2 and imaged in media containing 2 mM Ca^2+^ and verapamil. Cells expressing TrpC3-shRNA had a lower basal [Ca^2+^]_i_ level (Fig. 5*B,C and D*) than LacZ-shRNA-expressing cells. Additionally, cells expressing TrpC3-shRNA displayed a decreased amplitude of the [Ca^2+^]_i_ spike following PE-stimulation, and a decreased difference between peak and baseline [Ca^2+^]_i_ (Fig. 5*B,C and D*). This suggests that TrpC3 regulates both basal [Ca^2+^]_i _levels and also the transient [Ca^2+^]_i _elevation triggered by activation of α_1_-adrenergic receptors.

Another important type of [Ca^2+^]_i_ elevation in ventricular myocytes is the calcium rise that occurs with contraction. Previous studies have shown that hypertrophic gene expression is dependent on beat frequency and other aspects of beating-associated calcium, which are controlled in part by Ca_V_1.2 [14]. Therefore, we assayed contraction frequency and measured [Ca^2+^]_i_ in spontaneously beating myocytes to determine if TrpC3 affects beat frequency or beating-associated calcium elevations. We first measured intrinsic beat frequency in myocytes infected with lentiviruses encoding TrpC3-shRNA or LacZ-shRNA. Myocytes were plated at high density into multiple wells of a 96-well plate to produce synchronous beating of nearly all cells in a well. The cells were then subjected to 48 hours of PE stimulation to induce hypertrophy. We used phase-contrast microscopy to measure the contraction frequency of myocytes in 20 wells per condition, and found that TrpC3-shRNA did not have a significant effect on beat frequency (Fig 5*E*). We next measured [Ca^2+^]_i _by high-speed imaging (>80 Hz frame-rate) of Fluo4 calcium dye loaded into spontaneously beating myocytes that had been stimulated with PE for 72 hours. Compared to myocytes expressing LacZ-shRNA, myocytes expressing TrpC3-shRNA did not have significantly different beating-associated calcium dynamics (example traces shown in Fig. 5*F*). Analyzing the calcium traces of 23 cells expressing LacZ-shRNA and 23 cells expressing TrpC3-shRNA, we did not find large differences in beat frequency (0.25 Hz for LacZ-shRNA vs 0.27 Hz sec for TrpC3-shRNA; P = 0.89), the ratio of peak [Ca^2+^]_i_ to baseline [Ca^2+^]_i_, (5.7 vs 6.1; P = 0.51), or the time from baseline to peak [Ca^2+^]_i_ (0.21 sec vs 0.20 sec; P = 0.92). There may, however, be a small difference between LacZ- and TrpC3-shRNA expressing cells in the decay constant of the [Ca^2+^]_i _decline after a beat (0.67 sec vs 0.55 sec ; P = 0.07). Thus, while TrpC3 knockdown can decrease PE-induced [Ca^2+^]_i _elevations and decrease hypertrophy, it has little effect on contraction and beating-associated calcium dynamics. This suggests that the [Ca^2+^]_i _signals generated by TrpC3 are functionally uncoupled from the [Ca^2+^]_i _elevations generated by Ca_V_1.2 which lead to muscle contraction.

### TrpC3 localization is distinct from that of Ca_V_1.2

**Figure 6:**
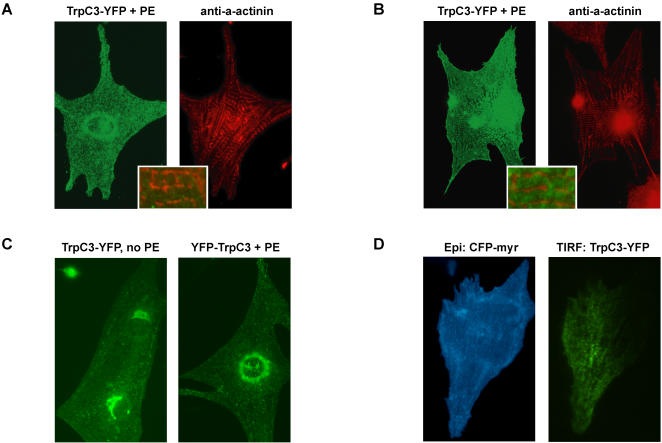
TrpC3 channel localization is distinct from that of Ca_V_1.2. (*A–B*) Epifluorescence microscopy images of fixed myocytes expressing TrpC3 C-terminally tagged with YFP (TrpC3-YFP). Cells were stimulated for 48 hours with PE to induce hypertrophy. The majority of cells display a punctate and perinuclear pattern of TrpC3-YFP (example shown in *A*, *left panel*). Co-staining with anti-α-actinin antibodies (*A*, *right panel*) reveals the localization of the Z-lines, which are closely apposed to the t-tubules. Magnifying a merge of the TrpC3-YFP and anti-α-actinin images (*A*, *inset*) shows little overlap between TrpC3-YFP puncta and the Z-lines. In some transfected myocytes, TrpC3-YFP puncta are completely excluded from the Z-lines (example shown in *B*, *inset*). (*C*) TrpC3-YFP expressed in a serum-starved, un-hypertrophied myocyte (*left panel*) is also distributed in puncta. The same punctate pattern is also observed in hypertrophied myocytes (*right panel*) expressing TrpC3 with an N-terminal YFP tag (YFP-TrpC3). (*D*) Live myocytes co-transfected with CFP-Myr (a marker of the plasma membrane) and TrpC3-YFP were imaged by epifluorecence and total internal reflection (TIRF) microscopy. CFP-Myr imaged by epifluorescence (*left panel*) illustrates the extent of the plasma membrane. A TIRF image of TrpC3-YFP (*right panel*) indicates that, in live myocytes, tagged TrpC3 is found in puncta within 200 nm of the cell surface.

One way in which Ca^2+^ signals generated by TrpC3 channels could be decoupled from those generated by Ca_V_1.2 is by being localized in different intracellular domains of myocytes. To determine the intracellular localization of TrpC3 channels, we used epifluorescence and total internal reflection (TIRF) microscopy to examine the distribution of tagged versions of TrpC3 channels in both unstimulated and hypertrophied myocytes. In the majority of hypertrophied myocytes, TrpC3 C-terminally-tagged with YFP (TrpC3-YFP) localized to puncta throughout the cell (example shown in Fig. 6*A*, *left panel*). We co-stained these cells with anti-α-actinin antibody, a marker of Z-lines, which are directly apposed to the t-tubules. The co-staining (Fig. 6*A*, *inset*) revealed little overlap between the localization of TrpC3 and that of Ca_V_1.2, which is mostly found in the t-tubules where it interacts with the contractile machinery. In a subset of myocytes (example shown in Fig. 6*B*), TrpC3-YFP was completely excluded from the Z-line, and thus isolated from Ca_V_1.2. The Trpc3-YFP punctate pattern was also observed in all transfected myocytes that had not undergone hypertrophy (Fig 3*C*, *left panel*), suggesting that the punctate distribution is not greatly affected by hypertrophy. We confirmed that a C-terminal YFP tag does not perturb the localization of TrpC3 by expressing TrpC3 with an N-terminal YFP tag and observing the same punctate distribution (Fig 3*C*, *right panel*). Finally, we employed TIRF microscopy to image live myocytes transfected with TrpC3-YFP. TIRF microscopy only illuminates fluorophores within 0.2 µm of the coverglass. The TIRF images revealed that in live myocytes TrpC3-YFP is in discrete puncta <200 nm from the cell surface (Fig. 6*D*, *right panel*). Thus, under multiple imaging and stimulation conditions, TrpC3 channel localization is distinct from that of the Ca_V_1.2 channels that regulate beating.

## Discussion

In this study we used an unbiased screen to identify ion channels that regulate cardiac hypertrophy. We designed a high-throughput assay using cultured neonatal rat ventricular myocytes to screen an RNAi library for calcium channels that regulate hypertrophy-associated genes. Using this approach we identified several Group 1 Trp channels, including TrpC3, that play an important role in the activation of genes associated with hypertrophy. This is the first example of an RNAi-based screen for genes that mediate cardiac hypertrophy and is a proof-of-principle for the utility of this general approach. We extended this RNAi screen with two other high-throughput assays for hypertrophy regulators: a real-time RT-PCR assay to identify genes dynamically regulated during hypertrophy and an automated microscopy assay to measure myocyte cell size. The widespread availability of genome-wide RNAi libraries will make it possible to use our screening methods to identify other genes that are required for the development and maintenance of cardiac hypertrophy and which may be novel targets for pharmaceutical development.

Our initial screens identified TrpC3 as a potent regulator of hypertrophy-associated gene expression. Reducing the expression of TrpC3 in a variety of ways reduced the expression of BNP and ANP in response to activation of α_1_-adrenergic receptors. We employed numerous controls for the specificity of RNAi knockdown of TrpC3, including the use of two RNAi methods, two TrpC3-shRNAs, two control LacZ-shRNAs, and successful rescue of TrpC3-shRNA's effect by overexpression of hTrpC3. Thus, while it is possible our TrpC3 RNAi affects hypertrophy due to non-specific knockdown of other targets (such as additional TrpCs), a great deal of evidence suggests the TrpC3-RNAi results are specific.

Interestingly, TrpC3 is involved in the induction of hypertrophy-associated genes in response to a wide variety of stimuli. This suggests that multiple receptors are linked to a common enzymatic pathway that leads to the activation of TrpC3 and to transcription of genes associated with cardiac hypertrophy. Notably, TrpC3 knockdown has a greater effect on BNP induction by PE than ET1, perhaps suggesting other TrpCs or other calcium currents may contribute differentially to different hypertrophic responses. Nonetheless, our finding that TrpC3 is a common element in the activation of gene expression in response to multiple stimuli suggests that it might be a useful target for the development of pharmacological blockers to treat heart disease.

Despite its key role in regulating hypertrophy-associated genes, TrpC3 does not have a strong effect on myocyte beat frequency or beating-associated calcium dynamics. Activation of TrpC3 by α_1_-adrenergic receptors leads to [Ca^2+^]_i_ elevation, but this Ca^2+^ does not seem to regulate beating calcium. This suggests that the Ca^2+^ that enters cells through TrpC3 channels is functionally isolated from Ca^2+^ elevations generated by Ca_V_1.2 channels that mediate muscle contraction. How does Ca^2+^ influx through these two channels differentially activate signaling pathways leading to different cellular events? One possibility is that each channel is associated with a distinct set of Ca^2+^-dependent signaling proteins that are activated by Ca^2+^ influx through that channel. For example, Ca_V_1.2 channels are localized close to, and efficiently activate, RyR2 channels, thereby triggering muscle contraction. In contrast, Ca^2+^ influx through TrpC3 apparently does not strongly activate RyR2 channels and therefore does not strongly affect beating. The observation that TrpC3 is especially efficient at activating gene expression suggests that it is associated with a different set of proteins than Ca_V_1.2. This is consistent with our observation that the intracellular distribution of TrpC3 is quite distinct from that of Ca_V_1.2 and RyR2. The dramatically different subcellular localization of TrpC3 and Ca_V_1.2 may explain why Ca^2+^ influx through TrpC3 channels does not affect beating calcium but can activate signaling pathways leading to the nucleus.

The finding that TrpC3 modulates hypertrophic gene expression but not beating does not imply that the calcium involved in beating cannot also affect hypertrophy. Indeed, calcium influx through Ca_V_1.2 has been implicated in both beating and hypertrophy. Previous reports have shown that pharmacological blockade of Ca_V_1.2 with dihydropyridines inhibits *in vitro* hypertrophy [14], a result confirmed by our RNAi results with Ca_V_1.2 (Fig. 1*E*). Thus, it is possible that the calcium involved in beating simultaneously regulates hypertrophy. According to the kinetic discrimination model, calcium-activated signaling molecules may convert changes in beating calcium dynamics into changes in the expression of hypertrophy genes. Alternatively, changes in beating calcium may activate hypertrophy through mechanical stretch pathways. It must now be determined if TrpC3 and Ca_V_1.2 activate hypertrophy by stimulating the same or alternate downstream pathways.

A surprising finding of this study is that TrpC3 knockdown has a strong effect on hypertrophic gene expression but does not prevent the increase in cell size associated with hypertrophy. This is unusual because nearly all genes that regulate hypertrophic gene expression also regulate cell size. A few genes, such as the transcription factor c-fos, however, affect hypertrophic gene expression without affecting cell size [25], providing some precedent for the effects of TrpC3. Future investigations of TrpC3 and c-fos may be thus useful in dissecting the pathways which differentially control the multiple phenotypes of hypertrophy. Additionally, because only a subset of the processes associated hypertrophy are deleterious [1], TrpC3 may be a uniquely interesting target for therapeutic intervention.

Therapeutic targeting of TrpC3 will likely require understanding the interplay of TrpC3 with other Trp channels. Our RNAi screen identified other Trp channels as regulators of hypertrophy, which we are currently following up. Additionally, Trp channel interactions may explain the curious result that TrpC3 slightly increases cell size in the absence of hypertrophic stimuli. Perhaps myocytes coordinately regulate the expression of TrpC3 and other Trps, such that knockdown of TrpC3 in the absence of other stimuli may modulate other Trp channels which regulate cell size. Such coordinate regulation of multiple Trp genes has been documented in other cell types [26]. In future work, it will be essential to assay the expression of other Trp channels after interventions targeting TrpC3 in the heart.

Our finding that TrpC3 is involved in regulating cardiac hypertrophy agrees with three recent studies that appeared during the preparation of this manuscript [27]–[29]. While those studies identified a role for TrpC3 in inducing hypertrophy, our study extends these initial findings in several important ways. First, we identified TrpC3 from an unbiased screen for ion channel genes that regulate cardiac hypertrophy, providing independent confirmation that TrpC3 mediates hypertrophy. Second, we have found that TrpC3 regulates gene expression independently of beat frequency. As TrpC3 is localized in a subcellular domain that is different from the localization of Ca_V_1.2, this provides strong evidence for the idea that Ca^2+^ influx through different ion channels differentially regulates contraction and hypertrophy in ventricular myocytes. Third, we show that TrpC3 is a common element in the signal transduction cascades activated by several agents that induce hypertrophy. Finally, we have found that TrpC3 strongly regulates hypertrophy-associated gene expression but has little effect on cell size, suggesting that these two pathways are at least partly independent. Two of the other recent papers found that overexpression of TrpC3 increased myocyte size [27], [28]. These studies indicate that some levels of TrpC3 perturbation, at least overexpression, are able to affect cell size. However, our results suggest that even strong TrpC3 knockdown has little effect on cell size while powerfully inhibiting hypertrophic gene expression. Taken together, the experiments in this manuscript add significantly to our understanding of the signaling cascades that lead to cardiac hypertrophy and identify a new potential target for the development of pharmaceutical agents to treat heart disease.

## Materials and Methods

### Molecular Cloning

We first compiled a database of the NCBI-curated sequences of the rat Group 1 Trp channels. A list of the NCBI accession numbers of the targeted genes is listed in Table S1. Diced-pools of short interfering RNA (d-siRNA) were prepared as described ([24]). Briefly, we prepared cDNA from cultured neonatal rat ventricular myocytes and rat E18 cortical neurons, using the RNEasy Mini Kit (QIAgen) and First-Strand Synthesis Kit (Invitrogen). For each Trp channel listed in Table S1, we performed the following sequence to create d-siRNA. We made a 400–500 base pair (bp) PCR product of the coding sequence of the target gene, using as template a 1∶1 mixture of the myocyte and cortical neuron cDNA. We then made a 400–500 bp nested PCR product from the initial PCR product. The nested PCR primers all possessed a short leader sequence (GCG) followed by the T7 promoter sequence (5′-TAATACGACTCACTATAGG-3′) and 18–21 gene specific nucleotides. Primer sequences are listed in Table S1. dsRNA was synthesized from the purified nested PCR products using the MEGAScript High Yield Transcription Kit (Ambion). dsRNA was treated with Dnase I at 37°C for 20 minutes, followed by denaturation at 75°C for 5 minutes and annealed at room temperature for 5 minutes to produce a dsRNA product of 400–500 bp. Concentrations of dsRNAs targeting the various Trp channels were normalized using a UV spectrophotometer. Then we used the BLOCK-iT Dicer RNAi Kit (Invitrogen) to perform the *in vitro* dicing reaction and purify the 21–23 nucleotide d-siRNA pools. We were able to successfully generate d-siRNA pools for all Group 1 Trp channel members, except TrpM1 and TrpM5. We were never able to obtain primary PCR products for TrpM1 and TrpM5 from the myocyte and cortical neuron cDNA, despite testing three different primer sets each. The repeated failure to PCR TrpM1 and TrpM5 suggests that these two genes are not expressed in myocytes and therefore not necessary for testing by knockdown in myocytes.

Short-hairpin RNAs (shRNAs) were first expressed from the lentiviral vector LentiLox 3.7 (LL37) [30], kindly provided by the laboratory of L. Attardi. shRNAs were cloned into LL37 using the method of Rubinson, *et al.* [30]. Briefly, we ordered a pair of oligonucleotides (Integrated DNA Technologies, Corallville, IA) that when annealed contained the shRNA coding sequence and appropriate ends for cloning into HpaI- and XhoI-cut LL37. We made two LL37-based constructs which each expressed an shRNA targeting different sequences within the rat TrpC3 coding sequence. One TrpC3-shRNA target sequence (termed TrpC3-shRNA#1: 5′-GTTTGCTCGTTCCAAACTC-3′) was chosen using the online target selection tool of Dharmacon, Inc. (http://www.dharmacon.com). The second TrpC3-shRNA target sequence (TrpC3-shRNA#2: 5′-GGCTGCGCATTGCCATAAA-3′) was designed as described [31]. The homologous sequence of TrpC3-shRNA#2 in *Homo sapiens* TrpC3 is 5′-GGCGGCGCACTGCCAGAAA-3′, where the underlined bases represent internal mismatches compared to the target sequence in rat TrpC3. The high number of internal mismatches makes hTrpC3 resistant to TrpC3-shRNA#2, and thus was used in RNAi rescue experiments. For negative controls, we also constructed two LL37-based vectors expressing shRNAs targeting two different sequences within the bacterial LacZ gene (LacZ-shRNA#1: 5′-GCACGTACCTGAATTTCGA -3′; LacZ-shRNA#2: 5′-TCGCTGATTTGTGTAGTCG-3′). The same shRNA target sequences were also cloned into the pSiren plasmid (BD Biosciences), using BamHI and EcoRI cloning sites and the oligonucleotide design method of the pSiren manual. For transfection experiments, we alternately used either pSiren- or LL37-based plasmids for testing the effects of shRNAs, and confirmed that each set of plasmids both worked in several of the assays. Comparisons between TrpC3-shRNA and negative control shRNA were always done using the same parent plasmid. For data shown, most luciferase assays employed LL37-based plasmids, while cell size assays were done using pSiren-based constructs. For most experiments, we employed TrpC3-shRNA#2 and LacZ-shRNA#2, but we verified that the other shRNAs produced similar results in multiple assays.

The BNP-Luciferase (BNP-Luc) construct, containing 1595 bp of the rat genomic sequence upstream of the BNP gene controlling the expression of firefly luciferase, was kindly provided by D. Gardner ([32]). ANP-Luciferase (ANP-Luc), containing 638 bp of the 5′ upstream flanking sequence of the rat ANP gene controlling firefly luciferase, was generously provided by C. Glembotski [33].

Human TrpC3 (hTrpC3) was cloned into pcDNA3 and kindly provided by D. Clapham. We subcloned hTrpC3 into the pCR8/GW/TOPO Gateway entry vector (Invitrogen). We then used the Gateway LR clonase reaction to subclone hTrpC3 into two Gateway-modified destination vectors, pCMV-XB-YFP and pcDNA3.1-Myc-His-B, generating constructs containing either TrpC3 with a C-terminal yellow fluorescent protein (YFP) tag, or TrpC3 with a C-terminal tag containing the Myc and 6x-His epitopes.

### Cell culture, transfection, and infection

Ventricles were dissected from 1-day-old Sprague-Dawley rats (Charles River), and dissociated using the neonatal rat NeoMyts Kit (Cellutron, Highland Park, NJ). Cells were pre-plated for two hours to separate adherent fibroblasts from non-adherent cardiac myocytes. Myocytes were resuspended in DMEM containing 10% fetal bovine serum (FBS), 2 mM L-glutamine, 5 mM 5-bromo-2-deoxyuridine (BrdU; Sigma), and penicillin/streptomycin (P/S) and plated onto either gelatin-coated coverglasses or culture dishes. Myocytes were plated at 700 cells/mm^2^ for cell size assays, and at 1100 cells/mm^2^ for other experiments.

For transfection experiments, myocytes were plated in 24-well plates that were either directly coated with gelatin (Sigma) or contained gelatin-coated coverglasses. The media was changed to OptiMEM (Invitrogen) 18–24 hours after plating the myocytes, and myocytes were transfected using 2 µl Lipofectamine 2000 per well. For luciferase experiments, 0.32 µg of firefly-luciferase plasmid, 0.08 µg of the constitutively-expressing Renilla plasmid pRLTK (Promega), and 0.4 µg of LL37- or pSiren-based plasmids were transfected per well. For overexpression of TrpC3 in luciferase assays, 0.8 µg of pcDNA3-based constructs were transfected. Four hours after transfection, the media was replaced with serum-starvation media containing DMEM, 2 mM glutamine, 5 mM BrdU, and P/S. Twenty-four hours later, fresh serum-starvation media was exchanged, and to designated cells we added 20 µM (R)-(-)-Phenylephrine hydrochloride (PE) (Sigma) to induce hypertrophy. A similar protocol was used for the induction of hypertrophy using 100 nM endothelin-1 (ET1; Sigma) or 150 ng/mL epidermal growth factor (EGF; Upstate Cell Signaling). Forty-eight to sixty hours later, myocytes were lysed for luciferase assays. Myocytes were fixed for immunocytochemistry and cell size assays 48 to 60 hours after initiation of PE stimulation.

Lentivirus was prepared as described ([34]). We performed a relative titer of LL37-TrpC3-shRNAs and LL37-LacZ-shRNAs, using myocyte infection and subsequent normalization of the concentrated viruses to make ensure each viral stock had the same number of infectious units per unit volume. For all experiments employing infection, infection was performed 36 hours after plating, adding virus sufficient to produce 60-70% infection and 4 µg/mL of polybrene (Sigma). Media was changed to plating media 8 hours later. For real-time PCR measurement of gene expression in infected myocytes, following infection the cells were placed in fresh plating media for 4 days before lysis. For beat-frequency measurements, cells were serum-starved (in 4∶1 DMEM:M199+1% Nutridoma-SP [Roche]) for 24 hours after completion of infection, stimulated with PE for 48 hours, and then subjected to beat frequency measurement.

### Real-time RT-PCR

Myocyte total RNA was isolated using the RNEasy Mini Kit (QIAgen), and cDNA to be used as template in real-time PCR was produced using the First-Strand Synthesis Kit (Invitrogen). Real-time PCR primers were either based on published sequences or designed using VectorNTI (Invitrogen). All real-time PCR primers used are listed in Table S2. Real-time PCR was conducted using the QuantiTect SYBR Green Kit (QIAgen) with a Stratagene Mx3000P real-time PCR machine. The ΔΔC_t_ method was used to calculate the relative difference of TrpC3 mRNA levels between samples, normalizing with GAPDH levels. Each real-time PCR reaction was repeated four times in parallel, and data are presented as the mean of the GAPDH-normalized values, with error bars representing standard error of the mean (SEM). At the end of each real-time PCR run, a dissociation curve was measured to show that only one PCR product was measured per reaction. The PCR products for each reaction were then run on a 3% agarose gel to confirm that the size of each product fit with the predicted size (a 150–250 bp PCR product). For the TrpC3, BNP, and GAPDH primer sets, the PCR products were also sent for sequencing to confirm that the proper target had been amplified.

### Luciferase assays

Myocytes were lysed using 1x Passive Lysis Buffer, and luciferase reactions were performed using the Dual-Luciferase Reporter Assay Kit (Promega). Reactions were performed and analyzed in a Veritas Microplate 96-well Luminometer (Turner Biosystems). The activity was measured both for firefly luciferase (encoded by BNP-Luc and ANP-Luc) and for Renilla luciferase (encoded by pRLTK). Renilla luciferase activity serves as an internal control. Data are presented as ratios of firefly:Renilla activity. Each condition was performed in at least six replicate wells, and each experiment was repeated on multiple occasions, with representative data shown in figures. Data are presented as mean plus SEM.

### Cell size

Cells were fixed for 15 minutes at room temperature in PBS containing 4% paraformaldehyde and 2% sucrose. Fixed cells were permeabilized with 0.25% Triton-X-100 in PBS for 10 minutes, blocked in 3% BSA in PBS for 30 minutes, and then incubated with anti-α-actinin antibody (Sigma, clone EA53) at 1∶500 for 1 hour at room temperature. After washing, we incubated with Alexa-488 goat anti-mouse secondary antibody (Molecular Probes/Invitrogen) at 1∶500 for 1 hour. Cells were then stained with Hoechst dye (Molecular Probes). For high-throughput cell size measurement, cells were plated in 24-well-plate Primaria culture dishes (Becton Dickinson), and left in PBS after Hoechst staining. For the traditional manually-measured cell size assay, cells were plated on coverglass and after fixing and staining were mounted on slides using AquaPolymount (Polysciences, Warrington, PA).

For high-throughput cell size analysis, cells were imaged using the 20x Objective of an Axon ImageXpress automated microscope (Axon Instruments). Images were acquired using filtersets to detect DsRed (expressed by pSiren), Alexa 488, and Hoechst. Six wells were used per condition, and 42 sites were imaged per well. An ImageXpress analysis script was composed that found and drew a border around transfected cells based on DsRed intensity, gated out transfected non-myocytes based on low intensity of Alexa 488 signal, and other artifacts (e.g., dead cells) were excluded using properties of all three filterset images. The script then output cell area, which after stringent gating, yielded approximately 1000 cell size measurements per condition. Data are presented as mean plus SEM.

To confirm the high-throughput cell size analysis, we used the traditional manually-measured cell size assay on cells transfected and treated identically as those in the high-throughput assay. Imaging was done by hand using a Nikon Eclipse TE2000-U epifluorescence microscope, 20x objective. Cell outlines were traced by hand and area was measured in OpenLab (Improvision, Lexington, MA). At least 150 cells were traced per condition. We also performed cell size analysis using the manual method on lentivirally-infected cells (data not shown).

### Beat Frequency Assay and Calcium Imaging

Myocytes were plated in 96 well plates and infected and cultured as described above. After 48 hours of PE stimulation, myocytes were taken out of 37°C incubator, and placed in Hanks′ Balanced Salt Solution (HBSS), supplemented with 0.1 % BSA, 1% Nutridoma-SP, and 20 µM PE, so that pH would remain stable but other stimulation conditions would resemble those in other experiments. Cells were imaged using phase-contrast and a 20x objective on a Nikon Eclipse TE2000-U to observe the synchronous beating of cells in each well. The number of beats in a 90 second period was counted manually. Data presented as mean plus SEM for 20 wells per condition.

For calcium imaging of beating-associated calcium, myocytes were cultured similarly as for the beat frequency assay, except they were cultured on 15 mm coverglass. The cells were then incubated in 2 µM Fluo-4-AM (Molecular Probes) in HBSS for 20 minutes at room temperature, washed twice in PBS, and then loaded into a closed perfusion chamber in room-temperature HBSS supplemented with 0.1 % BSA, 1% Nutridoma-SP, and 20 µM PE. Cells were imaged using a 60x objective on a Nikon Eclipse TE2000-U. Each cell was imaged for 25 seconds, at a frame rate of ∼80 Hz. Raw Fluo-4 traces were processed by background subtraction and then correction for photobleaching by dividing by an single-exponential curve fit to our experimentally-measured decay of Fluo-4 in each cell.

For calcium imaging of acute PE stimulation, the myocytes were infected with LL37-based lentiviruses two days after plating, or mock-infected, and then incubated 24 to 48 hours in serum-free media supplemented with Nutridoma-SP. The cells were then incubated in 2 µM fura-2-AM (Molecular Probes) in HBSS+0.1% BSA for 25 minutes at 37°C, washed twice in PBS, and then incubated in fresh HBSS for 10 minutes at 37°C. Cells were subsequently loaded into a closed perfusion chamber in room-temperature HBSS+containing 0.1% BSA and 10 µM of the Ca_V_1.2-blocker verapamil. For uninfected cells, the cells were imaged for 100 seconds, after which the appropriate stimulus was added: 20 µM PE+verapamil or 20 µM PE+verapamil+2 mM EGTA in calcium-free media. The cells stimulated in 2 mM Ca2+ solution were then stimulated 3 minutes later with media containing 2 mM Ca^2+^ and 65 mM K^+^ without verapamil, in order to determine the response to depolarization. For infected cells, after 100 seconds of imaging, HBSS containing 10 µM verapamil and 20 µM PE was added. Pairs of images (using excitation wavelengths of 340 nm and 380 nm) were taken every 5 seconds for a total duration of 700 seconds. Data were converted into ratios of the images taken with excitation wavelengths of 340 nm and 380 nm; these ratios are termed F340/F380. Ratio images were then converted to absolute calcium levels using a calcium calibration kit (Molecular Probes). Data are presented as mean of all cells measured plus SEM.

### Immunocytochemistry and TIRF imaging

Cells were fixed and permeabilized as described for cell size assays. Anti-α-actinin (Sigma) was used at 1∶500 and stained with 1∶500 goat anti-mouse Alex 594. Cells were imaged on a Nikon Eclipse TE2000-U epifluorescence microscope, 60x objective.

For the ANP-immunocytochemistry assay, myocytes were transfected with LL37-based plasmids and stimulated as described above. After fixation and permeabilization, cells were incubated overnight at 4°C with anti-ANP (Peninsula Labs, 1∶200) and anti-α-actinin (1∶500) antibodies, and subsequently stained with goat anti-rabbit Alexa 594 and goat anti-mouse Marina Blue secondary antibodies (Molecular Probes). Cells were imaged with the aforementioned microscope, 20x objective. The ANP levels of each cell were taken as the product of mean ANP-fluorescence per pixel multiplied by the area of the two-dimensional projection of the cell. Myocytes from three separate wells were used for each experiment, and data is presented as the mean ANP level for each condition, with error bars depicting SEM.

For TIRF imaging, cells were plated onto 15 mm coverglasses and transfected with the relevant constructs. CFP-Myr encodes CFP with a myristolation tag, and is used to indicate the plasma membrane in live cells. Myocytes co-transfected with CFP-Myr and TrpC3-YFP were put into a closed perfusion chamber containing room temperature HBSS+0.1% BSA. The cells were imaged with a 60x TIRF objective, imaging CFP in epifluorescence mode and YFP in TIRF mode.

### Western blot analysis

Human embryonic kidney 293 cells (HEK293) were transfected with hTrpcc3-MycHis using calcium phosphate. Two days after transfection, cells were lysed in lysis buffer (150 mM NaCl, 50 mM HEPES pH 7.5, 1% Triton-X-100, 0.4% sodium dodecyl sulfate [SDS]), resolved on an SDS-polyacrylamide gel, transferred to PVDF membrane (Invitrogen), and immunoblotted with anti-Myc primary antibody (Upstate Cell Signaling) followed by anti-mouse IgG conjugated to horseradish peroxidase (Pierce). Compared to untransfected HEK293 lysate, the transfected cells only displayed a single band that ran at ∼100 kDa.

### Statistical Analyses

Statistical tests were performed using Prism (GraphPad, San Diego, CA). For experiments involving three or more potential comparisons, we used one-way ANOVA followed by Dunnett's post-test. For experiments in which only one comparison was meaningful, unpaired one-tailed t-tests were employed.

## Supporting Information

Table S1 PCR primers used for the production of the d-siRNA library.
(0.08 MB DOC)

Table S2 PCR primers used in real-time RT-PCR
(0.03 MB DOC)
