# Supplementary material for: TrpC3 Regulates Hypertrophy-Associated Gene Expression without Affecting Myocyte Beating or Cell Size
Source: PLoS One. 2007 Aug 29;2(8):e802. doi: 10.1371/journal.pone.0000802 (PMC1950081; doi:10.1371/journal.pone.0000802)
Supplement: Table S1 — PCR primers used for the production of the d-siRNA library. (0.08 MB DOC) [file pone.0000802.s001.doc]

# Table S1: Primers used to create the d-siRNA library

| **Gene** | GenBank | **Primary PCR FORWARD primer** | **Primary PCR REVERSE primer** | **Nested PCR FORWARD primer** | **Nested PCR REVERSE primer** |
| --- | --- | --- | --- | --- | --- |
| TRPC1 | [NM_053558](http://www.ncbi.nlm.nih.gov/entrez/viewer.fcgi?val=NM_053558) | TGTCGTGGTTGTGATCGTGCTTAC | CTAATTTTTGGGATAAAACATAGC | GTAATACGACTCACTATAGGGGTAGCGATGCTTCATAAGAG | GTAATACGACTCACTATAGGGATATTTAGAAGTCCGAAAGCC |
| TRPC2 | [NM_022638](http://www.ncbi.nlm.nih.gov/entrez/viewer.fcgi?val=NM_022638) | GCTAGCAGGGCTCACCTCT | AGATCTGCAGAGTGCCCAAT | GCGTAATACGACTCACTATAGGTCTCTGCCAGGACTATGGCT | GCGTAATACGACTCACTATAGGGTGGTGAAGTATCGGCAGGT |
| TRPC3 | [NM_021771](http://www.ncbi.nlm.nih.gov/entrez/viewer.fcgi?val=NM_021771) | GCTGGCCAACATAGAGAAGG | TACTGAACCTTCGGTGGGAG | GCGTAATACGACTCACTATAGGGAGATCTGGAATCGGTGGAA | GCGTAATACGACTCACTATAGGTGAAGGCAGCAATGAAGATG |
| TRPC4 | [NM_080396](http://www.ncbi.nlm.nih.gov/entrez/viewer.fcgi?val=NM_080396) | AAATGTCAACGCCCCCTAC | CCTCGCAAGATCATTTCCAC | GCGTAATACGACTCACTATAGGACTTGAATGCCGTGGAAAAG | GCGTAATACGACTCACTATAGGGCTCTCTGGAACTCCGTGTC |
| TRPC5 | [AY064411](http://www.ncbi.nlm.nih.gov/entrez/viewer.fcgi?db=nucleotide&val=17887393) | GGAAGTTTGCGAGAACGAAG | CCCCGGATTTCACCTAACTC | GCGTAATACGACTCACTATAGGGGAGGCACAACTTGAGAAGC | GCGTAATACGACTCACTATAGGTCCTGCCACATAGAGTGCTG |
| TRPC6 | [NM_053559](http://www.ncbi.nlm.nih.gov/entrez/viewer.fcgi?val=NM_053559) | CCCTACCGATCCTCAGATCA | AGTCTCTCCCCAAGCCTTCT | GCGTAATACGACTCACTATAGGCAATCGCGGTGGTTTTAAGT | GCGTAATACGACTCACTATAGGTGTGCCAGCTGATTTCTGTC |
| TRPC7 | [XM_225159](http://www.ncbi.nlm.nih.gov/entrez/viewer.fcgi?db=nucleotide&val=34873769) | ATCCTTTTATTATCTCATAATGAG | CTAAATGTCCTTGCCCTTGTTCACC | GTAATACGACTCACTATAGGGGGCCTGGGGGCACTCGTG | GTAATACGACTCACTATAGGGAGGTCTCGAATGTGTCTG |
|  |  |  |  |  |  |
| TRPV1 | [NM_031982](http://www.ncbi.nlm.nih.gov/entrez/viewer.fcgi?db=nucleotide&val=14010882) | TTCTTCTTCCGAGGGATTCA | AACCAGGGCAAAGTTCTTCC | GCGTAATACGACTCACTATAGGCTGTACTTCAGCCAACGCAA | GCGTAATACGACTCACTATAGGTCCAGTTTACCTCGTCCACC |
| TRPV2 | [NM_017207](http://www.ncbi.nlm.nih.gov/entrez/viewer.fcgi?db=nucleotide&val=8394534) | GGAATTCTCAGGACCGTACC | CTCTAGGGAGGCATCCAGAA | GCGTAATACGACTCACTATAGGAAAGAACTCGGTGCTGGAGA | GCGTAATACGACTCACTATAGGCTACAGCAAAGCCGAAAAGG |
| TRPV3 | [BC089113](http://www.ncbi.nlm.nih.gov/entrez/viewer.fcgi?db=nucleotide&val=57921017) | ATGGAAGCCTGGTACAATCATCCTGC | GAGTTTGAAGATTTCATCTTCCTGG | GTAATACGACTCACTATAGGGCCCCAGTGAGGGAAAGACGCC | GTAATACGACTCACTATAGGGCCCGCAGTGTCTGGGTTCGAG |
| TRPV4 | [NM_023970](http://www.ncbi.nlm.nih.gov/entrez/viewer.fcgi?db=nucleotide&val=13027423) | ACTATGGCACTTACCGGCAC | TTGAACTTGCGAGACAGGTG | GCGTAATACGACTCACTATAGGGGTCGTAGAGAAGCAGCCAC | GCGTAATACGACTCACTATAGGGGGAGCACTTGAGAAGCAAC |
| TRPV5 | [NM_053787](http://www.ncbi.nlm.nih.gov/entrez/viewer.fcgi?db=nucleotide&val=31377509) | TCATTGAGCATGGAGCTGAC | CCACCTCCCCATTCATACTG | GCGTAATACGACTCACTATAGGTCCTACAGCCCAACAAAACC | GCGTAATACGACTCACTATAGGAAAGTAGCGAGAGGCACCAA |
| TRPV6 | [NM_053686](http://www.ncbi.nlm.nih.gov/entrez/viewer.fcgi?db=nucleotide&val=16758503) | TTCTGCAGATGGTTCCACAG | TGATCATCCCCTGAGGAGTC | GCGTAATACGACTCACTATAGGTCGCCTCTTCTTCTAGCTGC | GCGTAATACGACTCACTATAGGTGAGTCCTTGGTTATTGGGC |
|  |  |  |  |  |  |
| TRPM1 | NM_001037734 | NOT PRESENT IN CDNA |  |  |  |
| TRPM2 | [AY749166](http://www.ncbi.nlm.nih.gov/entrez/viewer.fcgi?db=nucleotide&val=58198287) | TGAATTCACGGAGTTTGTGG | CCCCGGATTTCACCTAACTC | GTAATACGACTCACTATAGGGGCACTGGGCCTTGCCTGGGGGC | GTAATACGACTCACTATAGGGCCTTTTGGAGGATCTTCTTG |
| TRPM3 | [XM_219902](http://www.ncbi.nlm.nih.gov/entrez/viewer.fcgi?db=nucleotide&val=34862087) | TACGCCAACTTTGGGGTGCCCGTG | GCTCTCAAAGCTATGAAAAGCCG | GTAATACGACTCACTATAGGGCAGCAGAATACACAAGTATTACAG | GTAATACGACTCACTATAGGGGTTCTAGACAGTCTTCTCATGG |
| TRPM4 | [NM_133607](http://www.ncbi.nlm.nih.gov/entrez/viewer.fcgi?db=nucleotide&val=25742848) | CCAGGACATACCCTCTGCTG | GCGTCCTCTCTGCTTCCTTA | GTAATACGACTCACTATAGGGGCCAACTGGCTGGTGGTGTTGC | GTAATACGACTCACTATAGGGCAGAGTGGCTAAGGGCCTCAGC |
| TRPM5 | [XM_344979](http://www.ncbi.nlm.nih.gov/entrez/viewer.fcgi?db=nucleotide&val=34861771) | NOT PRESENT IN CDNA |  |  |  |
| TRPM6 | [XM_219747](http://www.ncbi.nlm.nih.gov/entrez/viewer.fcgi?db=Nucleotide&dopt=GenBank&val=34861962) | GGGGCCTCCGCAAAGCCATGCGCG | CCTCTTGATTGTTTATCTTCCGG | GTAATACGACTCACTATAGGGCGTTATCAGCACCTGGTCTGAGG | GTAATACGACTCACTATAGGGGGTTTTATAACAGATGGATCTG |
| TRPM7 | [NM_053705](http://www.ncbi.nlm.nih.gov/entrez/viewer.fcgi?db=nucleotide&val=16758523) | AGGCTGTCGCAGAGTATTCC | AACCATGTCGCAGGACTTT | GCGTAATACGACTCACTATAGGCCTTCGTTCCTGTACCTCCA | GCGTAATACGACTCACTATAGGTCAAGTACCAGCAACTCCCC |
| TRPM8 | [NM_134371](http://www.ncbi.nlm.nih.gov/entrez/viewer.fcgi?val=NM_134371) | ACTTCGGAATCAGCTGGAGA | AGTTTGCCACCAACTTCCAG | GCGTAATACGACTCACTATAGGGTACATCTCTGAGCGCACCA | GCGTAATACGACTCACTATAGGGAACTTCTGCAGGTTGAGGC |
|  |  |  |  |  |  |
| TRPA1 | [NM_207608](http://www.ncbi.nlm.nih.gov/entrez/viewer.fcgi?db=nucleotide&val=46485384) | ATGTTCGTCCCAATTGTTCTCATGAAC | CTAGATGTCTGGGTGGCTAATAGAACAATG | GTAATACGACTCACTATAGGGACTGATTGGTTTGGCAGTTGG | GTAATACGACTCACTATAGGGTTAGTCTTAACTGCGTTTAAG |
|  |  |  |  |  |  |
| CaV1.2 | NM_012517 | CCTAGAGCAAGCCAATGAGG | AGGCCCTTCGACCTAGAGAG | GCGTAATACGACTCACTATAGG GAAGAGGACCAGCATGAAGC | GCGTAATACGACTCACTATAGG CGGGTCTCATCTGGAAACAT |
| LacZ |  | ATGAGTATTCAACATTTCCGTG | TTACCAATGCTTAATCAGTGAG | GTAATACGACTCACTATAGGG ATGAGTATTCAACATTTCCGTG | GTAATACGACTCACTATAGGGTTACCAATGCTTAATCAGTGAG |
