# Supplementary material for: TrpC3 Regulates Hypertrophy-Associated Gene Expression without Affecting Myocyte Beating or Cell Size
Source: PLoS One. 2007 Aug 29;2(8):e802. doi: 10.1371/journal.pone.0000802 (PMC1950081; doi:10.1371/journal.pone.0000802)
Supplement: Table S2 — PCR primers used in real-time RT-PCR (0.03 MB DOC) [file pone.0000802.s002.doc]

# Table S2: Primers used in real-time RT-PCR

| **Primer Name** | **Primer Sequence** |
| --- | --- |
| GAPDH Forward | 5'-TGCACCACCAACTGCTTAG-3' |
| GAPDH Reverse | 5'-GATGCAGGGATGATGTTC-3' |
| TrpC1 Forward | 5'-TTCCTCCCCATCCTCCTCATCG-3' |
| TrpC1 Reverse | 5'-CATTTCTCCCAAGCACATCTACGC-3' |
| TrpC2 Forward | 5'-CGAATCAACACCTACCGGGGCA-3' |
| TrpC2 Reverse | 5'-TGATTCCGGCACATGCCCAG-3' |
| TrpC3 Forward | 5’-TCAATCAGCCAACACGATATCAGCA-3’ |
| TrpC3 Reverse | 5’-TTCCTCCGTCGCTTGGCTCTTAT-3' |
| TrpC4 Forward | 5'-AAGGATTAGCTTCACGGGGTG-3' |
| TrpC4 Reverse | 5'-CCTCCTCCTGGGCGTGTTTC-3' |
| TrpC5 Forward | 5'-TGAGTCGTCAGGCAAACGGTC-3' |
| TrpC5 Reverse | 5'-TCCTGCCACATAGAGTGCTGC-3' |
| TrpC6 Forward | 5'-AGAAATTTGGAATTTTGGGAAG-3' |
| TrpC6 Reverse | 5'-TCCTTATCAATCTGGGCCTGC-3' |
| TrpC7 Forward | 5'-CCTGTACTCCTACTACCGAGGTGC-3' |
| TrpC7 Reverse | 5'-TGGTGACATTATAAACGCCGTAC-3' |
